# MCM-YOLO: A Lightweight Conflict Mitigation Network for Industrial Metal Surface Defect Detection

**DOI:** 10.3390/s26144349

**Published:** 2026-07-09

**Authors:** Shuhao Zhang, Kunjin He, Jiachen Xu, Heshan Sha, Zhengming Chen

**Affiliations:** 1 College of Information Science and Engineering, Hohai University, Changzhou 213200, China; shuhaozhang@hhu.edu.cn (S.Z.); jiachenxu@hhu.edu.cn (J.X.); heshansha@hhu.edu.cn (H.S.); 19871348@hhu.edu.cn (Z.C.); 2 Key Laboratory of Maritime Intelligent Cyberspace Technology of Ministry of Education, Hohai University, Changzhou 213200, China

**Keywords:** metal surface defect detection, lightweight neural networks, YOLO, multiscale feature fusion, attention mechanisms

## Abstract

Industrial metal surface defect detection is essential for visual sensing-based quality inspection, where lightweight models must balance reliable defect perception and efficient deployment under limited computational resources. However, weak textures, blurred boundaries, and small defect scales often reduce the reliability of lightweight detectors, while heavy global modeling modules increase computational cost. This article proposes a lightweight You Only Look Once (YOLO)-based framework, termed Mediation-based Conflict Mitigation YOLO (MCM-YOLO), for industrial metal surface defect detection. Built upon YOLOv11, MCM-YOLO introduces a C3-Res2Lite (C3-R2L) block to enhance fine-grained local representation and an Aggregated Bidirectional Feature Pyramid Network (Agg-BiFPN) to strengthen multiscale feature aggregation. We further observe that directly coupling the enhanced backbone and strengthened neck may degrade performance, which is referred to as Feature Integration Conflict (FIC). To alleviate the potential feature incompatibility associated with FIC, this article introduces a synergistic attention block, termed MCM-SAB, at the critical interface to perform coordinated channel recalibration and spatial refinement. Experiments on NEU-DET and GC-10 show that MCM-YOLO achieves 80.4% and 65.1% mean average precision (mAP) at an intersection-over-union (IoU) threshold of 0.5, respectively, with 6.3 giga floating-point operations (GFLOPs) and 2.70 million parameters. These results indicate that MCM-YOLO provides a competitive accuracy–efficiency tradeoff for industrial visual inspection.

## 1. Introduction

With the continuous advancement of intelligent manufacturing and Industry 4.0, industrial product quality control is rapidly evolving from offline sampling inspection toward online, intelligent, and closed-loop paradigms. In critical scenarios involving metal materials, castings, and plates, surface defect detection not only directly affects product yield but is also closely related to online visual sensing and closed-loop quality control in industrial systems [1,2,3]. Compared with general object detection tasks, industrial metal surface defects usually exhibit characteristics such as small scale, limited samples, low contrast, texture similarity, and diverse morphologies [4]. Meanwhile, real-world production-line deployment imposes strict requirements on the real-time performance and stability of detection algorithms, requiring models to achieve both high-throughput inference and low missed-detection risk under the constrained computational resources of edge devices [5,6]. Therefore, improving the perception, representation, and discrimination capabilities of lightweight models for complex defect signals under the constraints of industrial online visual sensing has become an important research issue in industrial visual sensing data processing.

Before the widespread adoption of deep learning, industrial defect detection mainly relied on handcrafted features and traditional image processing, such as color-statistical analysis [7], Gabor filtering [8], local regularity measures [9], and adaptive thresholding [10]. Although these methods are physically interpretable, their performance heavily depends on scene priors and imaging conditions, making them less robust under complex backgrounds, illumination variations, and cross-scene transfer settings.

In recent years, convolutional neural network (CNN)-based object detection methods have significantly advanced industrial defect detection and have gradually evolved into several technical paradigms, including two-stage and one-stage detectors. Two-stage methods, represented by the R-CNN family and its variants, such as Faster R-CNN [11] and Mask R-CNN [12], achieve high detection accuracy through region proposal generation and region of interest (RoI)-level refined prediction, and have been applied to metal-related industrial defect scenarios such as steel surfaces [13] and rail surfaces [14]. In contrast, one-stage detectors such as Single Shot MultiBox Detector (SSD) [15], RetinaNet [16], and the You Only Look Once (YOLO) series [17] provide end-to-end prediction and higher inference efficiency, making them more suitable for engineering tasks with stringent real-time requirements. For example, Ma et al. [18] applied SSD to the recognition and localization of concrete surface quality defects, while Yang et al. [19] introduced deformable convolution and an improved feature fusion structure into RetinaNet to improve steel surface defect detection performance. However, these representative deep detection frameworks may still be constrained by insufficient local representation capability and limited multiscale fusion quality when dealing with fine-scale defects, weak textures, and complex backgrounds.

Under the constraints of low latency and practical deployability in industrial online inspection, the YOLO series has become one of the most commonly used baselines for industrial surface defect detection, owing to its end-to-end one-stage framework, mature multiscale feature fusion mechanism, and relatively stable engineering implementation. For instance, Zhou et al. [20] introduced convolution and attention enhancement mechanisms into YOLOv8 to improve steel surface defect detection. Shen et al. [21] enhanced the multiscale representation capability of YOLOv5 for printed circuit board assembly (PCBA) defects by improving the spatial pyramid pooling (SPP) structure and incorporating large-kernel convolution. Wang et al. [22] enhanced YOLOv10n for small-defect detection in photovoltaic modules through wavelet-based feature extraction and adaptive multiscale feature fusion. Fan et al. [23] proposed a dual-branch YOLOv11-based network for industrial inspection, in which scale-specific branches were designed to improve the perception of large and small defects under complex backgrounds. However, when metal surface defects are characterized by weak textures, blurred boundaries, small targets, and low contrast, lightweight convolutional detectors may still suffer from insufficient representation capability, leading to missed detections, localization deviations, or category confusion [24].

To address this limitation, some studies have attempted to introduce global modeling mechanisms such as Transformers to enhance contextual dependency modeling. For example, Gao et al. [25] and Liu et al. [26] proposed improved networks based on Swin Transformer and dual-branch global context modeling, respectively. Chen et al. [27] further improved steel defect detection through hierarchical CNN–Transformer feature interaction, while Shang et al. [28] introduced a defect-aware Transformer network with defect-aware modeling and graph positional encoding for complex surface defect scenarios. However, such global modeling modules usually incur higher computational and memory costs, thereby posing greater challenges for low-latency implementation on edge devices or in resource-constrained online deployment scenarios [29]. As a result, industrial metal surface defect detection currently faces a typical dilemma: relying solely on lightweight convolutional networks may be insufficient to characterize complex defect patterns, whereas directly introducing heavy global modeling modules substantially compromises deployment efficiency.

A natural way to alleviate this dilemma is to enhance fine-grained local texture representation in the backbone while improving cross-scale feature aggregation in the neck. However, our experiments show that these two enhancements do not always produce additive gains when they are directly combined. Specifically, although the lightweight backbone enhancement and the strengthened neck structure are individually beneficial, their direct coupling may lead to non-additive performance improvement or even performance degradation. In this article, we refer to this empirically observed phenomenon as Feature Integration Conflict (FIC). Here, FIC is used as an explanatory term for the possible incompatibility between locally sensitive backbone features and aggregation-oriented neck inputs in terms of semantic level, channel-response distribution, and spatial activation patterns. This observation suggests that a lightweight interface mediation mechanism may be beneficial before dense multiscale aggregation.

To this end, this article proposes a lightweight defect detection framework for industrial metal surface defect detection, termed Mediation-based Conflict Mitigation YOLO (MCM-YOLO). Built upon YOLOv11, MCM-YOLO introduces a C3-Res2Lite (C3-R2L) block into the backbone to enhance fine-grained local representation and constructs an Aggregated Bidirectional Feature Pyramid Network (Agg-BiFPN) in the neck to improve multiscale feature aggregation. Meanwhile, to alleviate the potential feature incompatibility caused by directly coupling the enhanced backbone and strengthened neck, a lightweight synergistic attention block, termed MCM-SAB, is inserted at the critical feature interface. By combining Efficient Channel Attention (ECA)-based channel recalibration and Simple Parameter-Free Attention Module (SimAM)-based spatial refinement, MCM-SAB is designed to improve feature compatibility before dense multiscale aggregation. With these designs, MCM-YOLO aims to improve local defect representation, multiscale feature fusion, and cross-module feature coordination while maintaining a lightweight accuracy–efficiency tradeoff. The main contributions of this article are summarized as follows.

1. A lightweight fine-grained feature extraction block, termed C3-R2L, is proposed to enhance the representation of weak textures, slender structures, and blurred boundaries in small-scale metal surface defects with low computational overhead.
2. An aggregated cross-scale feature fusion structure, termed Agg-BiFPN, is designed to strengthen multiscale semantic interaction and improve feature aggregation quality under complex backgrounds and scale-varying defect scenarios.
3. A non-additive performance phenomenon is observed when the enhanced backbone and strengthened neck are directly coupled, and this phenomenon is cautiously described as FIC to analyze possible feature incompatibility at the backbone–neck interface. Based on this observation, MCM-SAB is proposed to perform lightweight interface mediation by combining the channel recalibration capability of ECA with the spatial refinement capability of SimAM.
4. A complete lightweight industrial metal surface defect detection framework, namely MCM-YOLO, is constructed and evaluated on NEU-DET and GC-10. Experimental results show that the proposed method achieves a competitive tradeoff among detection accuracy, model complexity, and inference efficiency.

## 2. Related Work

### 2.1. YOLOv11 Baseline

**Figure 1:**
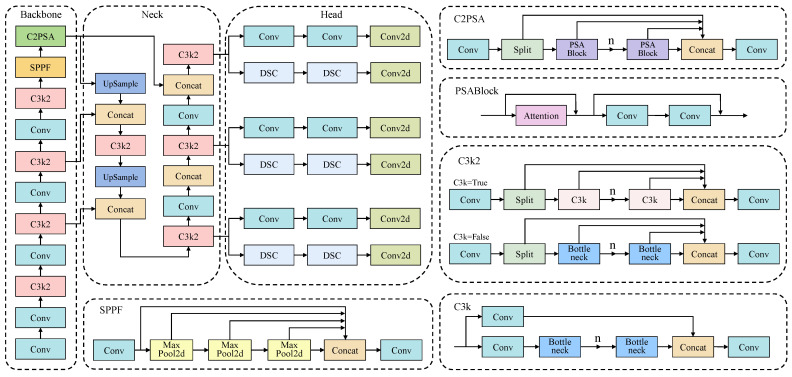
Overall architecture of YOLOv11.

As a representative one-stage object detection framework, YOLOv11 [30] achieves a favorable balance among detection accuracy, inference speed, and practical deployability, and therefore serves as a strong baseline for lightweight detection tasks. As shown in Figure 1, YOLOv11 follows the standard backbone–neck–head architecture. Specifically, the backbone employs C3k2 modules for efficient feature encoding, incorporates spatial pyramid pooling-fast (SPPF) to enlarge the receptive field, and introduces the C2PSA module to enhance feature representation. The neck follows the design of the Path Aggregation Network (PANet), constructing bidirectional top-down and bottom-up information flows for multiscale feature fusion. The head adopts a decoupled design for classification and bounding-box regression, thereby improving localization and recognition of multiscale targets.

However, for challenging metal surface defect samples characterized by small scales, weak textures, and blurred boundaries, the standard backbone still has limited capacity to capture fine-grained texture variations. Meanwhile, although the original neck provides multiscale fusion capability, its cross-level interaction density and aggregation efficiency still leave room for improvement under large defect-scale variations.

### 2.2. Feature Fusion and BiFPN

**Figure 2:**
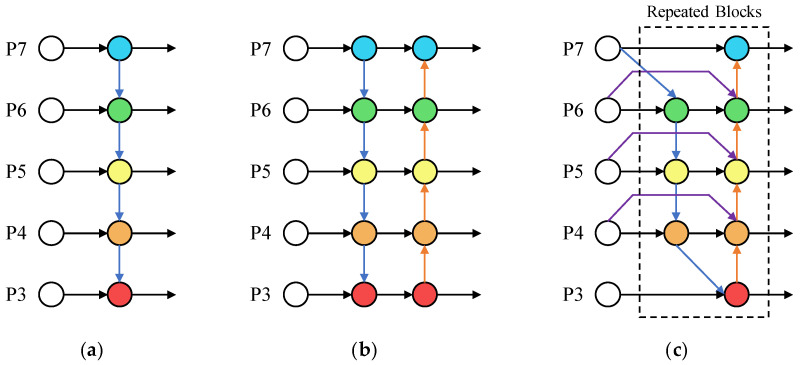
Multiscale feature fusion architectures: (**a**) FPN, (**b**) PANet, (**c**) BiFPN.

Multiscale feature fusion is a key component in object detection for handling target scale variations. As shown in Figure 2, the Feature Pyramid Network (FPN) [31] transfers high-level semantic information to low-level features through a top-down pathway, thereby enhancing the semantic representation of shallow features. PANet [32] further introduces a bottom-up pathway, strengthening the feedback of localization information and shallow details. On this basis, BiFPN [33] enables more effective information interaction among features at different scales by constructing an efficient bidirectional cross-level connection structure. In addition to topology optimization, another core advantage of BiFPN lies in its fast normalized weighted feature fusion mechanism. This mechanism assigns learnable weights to different input branches and performs adaptive feature aggregation through normalization, thereby reducing information redundancy and contribution imbalance caused by simple concatenation or direct summation. Therefore, it is well suited for detection scenarios involving small targets, weak textures, and complex backgrounds.

Nevertheless, for industrial metal surface defect detection, where target scales vary widely and local anomaly cues are sensitive to feature degradation, the fixed bidirectional fusion topology may not fully exploit the complementary information among different feature levels. This limitation motivates a more aggregation-oriented feature fusion design.

### 2.3. Lightweight Attention Mechanisms

Attention mechanisms can enhance the network’s focus on critical anomalous regions through adaptive recalibration of feature responses. In industrial metal surface defect detection tasks, defect regions are usually small, low in contrast, and easily disturbed by background textures. Therefore, lightweight yet effective attention modeling is important for improving fine-grained anomaly responses.

**Figure 3:**
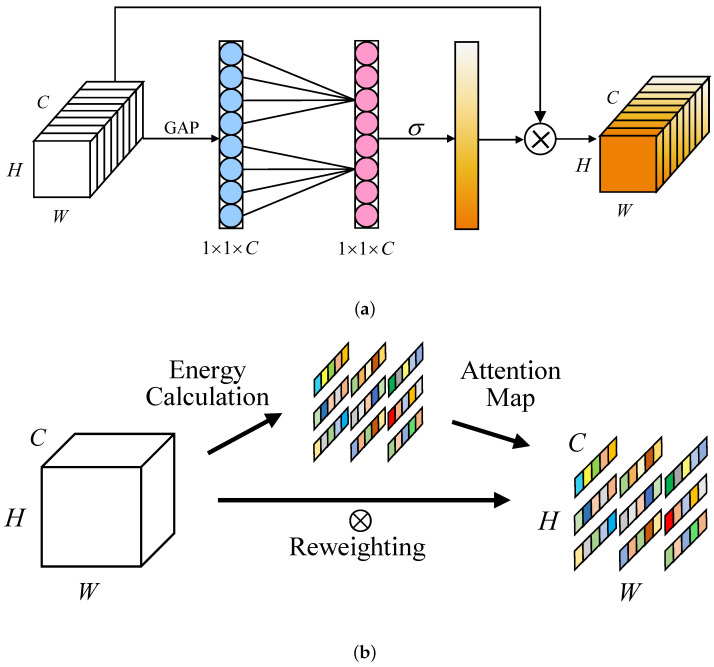
Structures of the lightweight attention mechanisms: (**a**) ECA, (**b**) SimAM.

ECA [34] is an efficient channel attention mechanism. As shown in Figure 3a, ECA first compresses the spatial dimensions through global average pooling and then directly models cross-channel dependencies using a local 1D convolution to generate channel reweighting coefficients. Compared with conventional channel attention methods that rely on dimensionality reduction and fully connected mappings, ECA can provide accurate channel recalibration with very low parameter overhead while avoiding channel information loss.

In contrast, SimAM [35] is a parameter-free attention mechanism inspired by neuroscience. As shown in Figure 3b, SimAM evaluates the saliency of feature responses by constructing an energy function and generates 3D attention weights without introducing additional learnable parameters. Through element-wise reweighting, it enhances responses to subtle local anomaly regions while introducing minimal additional model complexity.

The complementary properties of ECA and SimAM make them suitable candidates for lightweight channel-level and neuron-level feature refinement, providing the basis for the interface mediation module introduced in Section 3.

## 3. Proposed Method

### 3.1. Overall Architecture of MCM-YOLO

To address insufficient fine-grained representation, limited multiscale interaction, and unstable cross-module feature alignment in industrial metal surface defect detection, this article proposes a lightweight detection framework, termed MCM-YOLO. The name MCM-YOLO is a self-defined designation. Specifically, “MCM” denotes “Mediation-based Conflict Mitigation”, which reflects the central design rationale of this framework: a lightweight mediation mechanism is introduced to alleviate the potential feature integration conflict that may occur between the enhanced backbone and the strengthened neck. Therefore, MCM-YOLO does not refer to a single independent module, but to the complete YOLO-based detection framework that integrates fine-grained backbone enhancement, aggregated multiscale feature fusion, and interface-level feature mediation. Built upon YOLOv11, MCM-YOLO preserves the overall backbone–neck–head detection paradigm while introducing targeted improvements from three aspects, namely feature extraction, feature fusion, and cross-module interface mediation.

**Figure 4:**
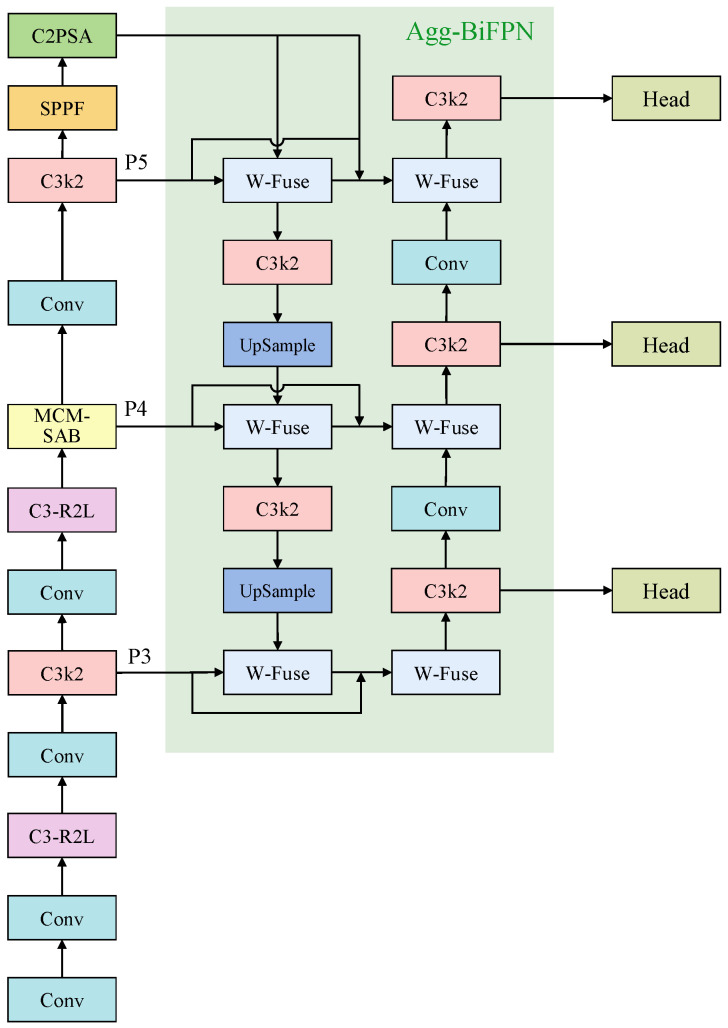
Overall architecture of MCM-YOLO.

The overall architecture of MCM-YOLO is illustrated in Figure 4. Given an input defect image $I\in R^{H\times W\times 3}$, its forward feature flow mainly involves the following three stages. First, in the backbone, the first and third standard C3k2 blocks are replaced with the proposed C3-R2L block to enhance fine-grained local representation at different depths and provide more discriminative multiscale inputs for subsequent feature fusion. Second, in the neck, Agg-BiFPN is constructed to strengthen bidirectional cross-scale interaction and multi-source feature aggregation. Finally, MCM-SAB is inserted at the P4 feature level, where cross-module interaction is relatively concentrated, to recalibrate interface features before dense multiscale aggregation. Through these designs, MCM-YOLO aims to improve local defect representation, multiscale feature fusion, and cross-module feature compatibility while maintaining a lightweight structure.

To clarify the architectural design, Table 1 summarizes the functional roles of the proposed components in MCM-YOLO. Specifically, C3-R2L, Agg-BiFPN, and MCM-SAB are designed with complementary roles, focusing on local representation enhancement, multiscale feature aggregation, and interface-level mediation, respectively.

### 3.2. C3-R2L Block for Fine-Grained Feature Extraction

The C3-R2L block is introduced into the backbone of YOLOv11 to enhance fine-grained local representation for metal defect patterns while keeping the additional computational cost low.

**Figure 5:**
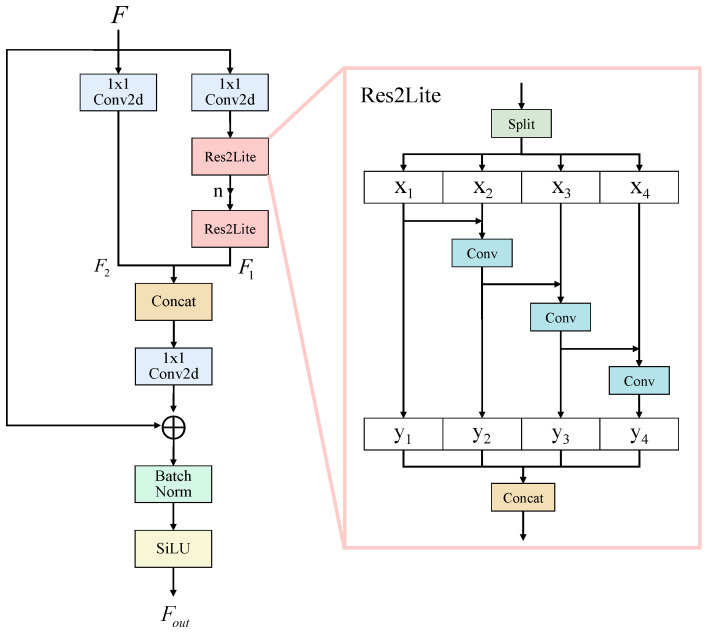
Structure of the C3-R2L block.

As shown in Figure 5, the C3-R2L block adopts a dual-branch structure. Given an input feature tensor $F\in R^{C\times H\times W}$, the input is first split by two parallel 1×1 convolution branches. The first branch feeds the features into *n* cascaded Res2Lite bottleneck units to extract local multiscale representations, while the second branch performs only a lightweight mapping to preserve the original information flow and basic texture information. The outputs of the two branches are then concatenated along the channel dimension and transformed by an output mapping to generate the fused feature. This process can be formulated as

(1) $$F_{1}=M\left({\mathrm{Conv}}_{1}\left(F\right)\right),\;F_{2}={\mathrm{Conv}}_{2}\left(F\right),$$

where ${\mathrm{Conv}}_{1}\left(\cdot \right)$ and ${\mathrm{Conv}}_{2}\left(\cdot \right)$ denote the 1×1 convolution mappings in the two branches, respectively, and M(·) denotes the deep transformation function composed of *n* cascaded Res2Lite units.

To reduce the information loss that may be caused by deep transformations, a residual shortcut is further introduced between the input and output. When the input and output dimensions are consistent, the module output can be written as

(2) $$F_{out}=\mathrm{SiLU}\left(\mathrm{BN}\left({\mathrm{Conv}}_{3}\left(\left[F_{1},F_{2}\right]\right)+F\right)\right),$$

where [·,·] denotes channel-wise concatenation, and ${\mathrm{Conv}}_{3}\left(\cdot \right)$ denotes the output 1×1 convolution mapping. This residual design helps improve the stability of feature transmission. Compared with the standard C3k2 block, the C3-R2L block is designed to better preserve local high-frequency texture information while maintaining low additional overhead.

The core component of the C3-R2L block is the Res2Lite unit. This unit follows the hierarchical residual multiscale representation idea of Res2Net [36]. Specifically, it divides the input into several groups of subfeatures along the channel dimension and constructs equivalent receptive fields at different scales through progressive local convolution interactions. Considering the lightweight requirements under edge deployment conditions, Res2Lite applies a moderate simplification to the standard Res2Net structure to reduce redundant computational branches.

Assume that the input feature of a Res2Lite unit is evenly split into *s* groups of subfeatures $\left\{x_{1},x_{2},\ldots ,x_{s}\right\}$ along the channel dimension. In this article, the default split scale is set to s=4. Then the progressive feature transformation inside Res2Lite can be expressed as

(3) $$y_{1}=x_{1},\;y_{i}=G_{i}\left(x_{i}+y_{i-1}\right),\;i\in \left\{2,3,\ldots ,s\right\},$$

where $G_{i}\left(\cdot \right)$ denotes the lightweight local feature extraction operation on the *i*-th data flow, consisting of a 3×3 convolution, BatchNorm, and SiLU activation. Finally, the responses of all groups are concatenated again along the channel dimension, yielding the output of Res2Lite as

(4) $$Y_{r}=\left[y_{1},y_{2},\ldots ,y_{s}\right].$$

In this way, C3-R2L integrates hierarchical local multiscale modeling into a lightweight dual-branch structure, which helps improve the backbone’s sensitivity to subtle texture variations and local anomalous patterns.

### 3.3. Agg-BiFPN for Cross-Scale Feature Aggregation

To enhance cross-scale feature aggregation in the neck, this article constructs an aggregated feature pyramid network, termed Agg-BiFPN, based on BiFPN. While preserving bidirectional feature propagation, Agg-BiFPN introduces denser cross-scale connections at key nodes, especially intermediate-scale nodes, allowing them to receive and integrate multi-source features from different levels. This design improves the coordination between shallow detail information and deep semantic information.

**Figure 6:**
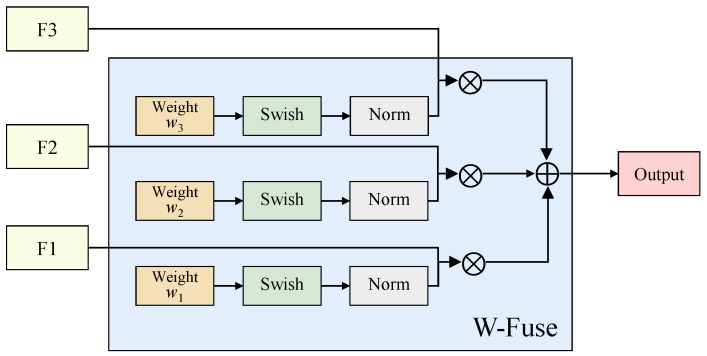
W-Fuse weighted aggregation mechanism.

To adaptively model the relative importance of different input branches, W-Fuse, an adaptive weighted aggregation operator, is introduced into the key aggregation nodes of Agg-BiFPN. As shown in Figure 6, W-Fuse assigns learnable weights to multiple input features, obtains adaptive coefficients through Swish activation and normalization, and then performs weighted summation to produce the fused output. Compared with direct summation or concatenation, W-Fuse enables the network to adjust the contribution of each input branch according to its relative importance at the current node.

Assume that a fusion node receives *N* feature inputs, and the corresponding set of learnable weight parameters is $\left\{w_{1},w_{2},\ldots ,w_{N}\right\}$. Then, the normalized weight ${\tilde{w}}_{i}$ for the *i*-th input branch can be expressed as

(5) $${\tilde{w}}_{i}=\frac{\mathrm{Swish}(w_{i})}{\sum_{j=1}^{N}\mathrm{Swish}\left(w_{j}\right)+\epsilon },$$

where Swish(·) is adopted to obtain smoothly varying learnable weights before normalization, and ϵ is a stabilizing term introduced to prevent division by zero; in this article, ϵ is set to 0.0001. After normalization, different input branches obtain adaptive weight distributions according to their relative importance.

Furthermore, the weighted aggregation process for multiple input features can be expressed as

(6) $$O=\sum_{i=1}^{N}{\tilde{w}}_{i}\cdot \mathrm{Resize}\left(I_{i}\right),$$

where $I_{i}$ denotes the original feature of the *i*-th input branch, Resize(·) denotes the scale alignment operation that resizes the feature to the target node in terms of spatial resolution and channel dimension, such as upsampling or downsampling, and *O* denotes the final fused output. Through this strategy, Agg-BiFPN enables flexible multi-source interaction across feature levels and reduces contribution imbalance during feature fusion.

### 3.4. MCM-SAB for Feature Conflict Mitigation

Although C3-R2L and Agg-BiFPN are designed to improve different aspects of defect detection, directly cascading them does not always lead to additive performance gains. As observed in the ablation study in Section 4.5.1, the two modules are individually beneficial, whereas their direct combination may result in non-additive improvement or even performance degradation. In this article, this phenomenon is referred to as FIC. It should be emphasized that FIC is used as an empirically motivated explanatory concept rather than a fully proven theoretical mechanism. It does not refer to tensor-size incompatibility, because the feature maps are resized and channel-aligned before fusion in Agg-BiFPN. Instead, it describes the possible incompatibility between locally sensitive backbone features and aggregation-oriented neck inputs in terms of semantic level, channel-response distribution, and spatial activation patterns.

From the perspective of feature representation, C3-R2L tends to strengthen local texture-sensitive responses, which is beneficial for weak-texture and small-scale defects. In contrast, Agg-BiFPN performs dense multi-source feature aggregation and is expected to benefit from input features with relatively stable semantic and response distributions. When these two types of representations are directly coupled at intermediate feature levels, especially around the P4 interface, some defect-related cues may be weakened, dispersed, or biased toward background textures during feature aggregation. This interpretation is not based solely on the network structure, but is further examined in Section 4 through component ablation, convergence analysis, module-overhead comparison, lightweight interface mediation comparison, insertion-position analysis, and GT-guided Grad-CAM analysis.

Motivated by this observation, MCM-SAB is introduced as a lightweight interface mediation module. MCM-SAB aims to improve feature compatibility before dense multiscale aggregation by sequentially applying ECA-based channel recalibration and SimAM-based spatial/neuron-level refinement.

**Figure 7:**
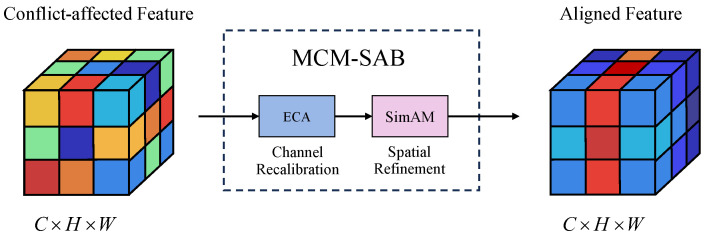
MCM-SAB for feature conflict mitigation.

As shown in Figure 7, MCM-SAB sequentially refines interface features by first performing channel recalibration and then spatial refinement. Let the interface input feature tensor be $X\in R^{C\times H\times W}$. MCM-SAB first generates channel attention weights through ECA and recalibrates the input feature along the channel dimension to obtain the intermediate feature $X^{\prime }$ as

(7) $$X^{\prime }=M_{c}\left(X\right)⊗X,$$

where $M_{c}\left(X\right)\in R^{C\times 1\times 1}$ denotes the channel attention weights dynamically generated by ECA from the input feature *X*, and ⊗ denotes element-wise multiplication with broadcasting along the spatial dimensions. This process helps suppress redundant channel responses and enhance effective semantic channels related to defects.

On this basis, SimAM further performs 3D neuron-level reweighting on the channel-recalibrated feature $X^{\prime }$ to obtain the final refined output feature *Y* as

(8) $$Y=M_{s}\left(X^{\prime }\right)⊗X^{\prime },$$

where $M_{s}\left(X^{\prime }\right)\in R^{C\times H\times W}$ denotes the 3D spatial attention weights generated by SimAM from $X^{\prime }$ based on the energy function. This operation helps enhance the network’s response to more discriminative defect regions and alleviate the potential spatial deviation associated with interface mismatch. Through the above two-stage synergistic mechanism, MCM-SAB implements a sequential refinement process from channel recalibration to spatial refinement, thereby improving the representation quality of interface features.

Considering that the P4 node lies in the transition region between shallow high-frequency textures and deep semantic information, it serves as an important interface where the enhanced backbone features are delivered to the strengthened neck for dense aggregation. Therefore, this article deploys MCM-SAB at this node. Let its original input feature be $X_{P4}$. Then, the refined output can be expressed as

(9) $$Y_{P4}=F_{\text{MCM-SAB}}\left(X_{P4}\right),$$

where $F_{\text{MCM-SAB}}\left(\cdot \right)$ denotes the synergistic mapping function of MCM-SAB. By performing lightweight channel recalibration and spatial/neuron-level refinement at this critical interface, MCM-SAB is expected to provide more compatible intermediate features for subsequent multiscale aggregation with only a small additional cost.

## 4. Results and Discussion

### 4.1. Datasets

**Figure 8:**
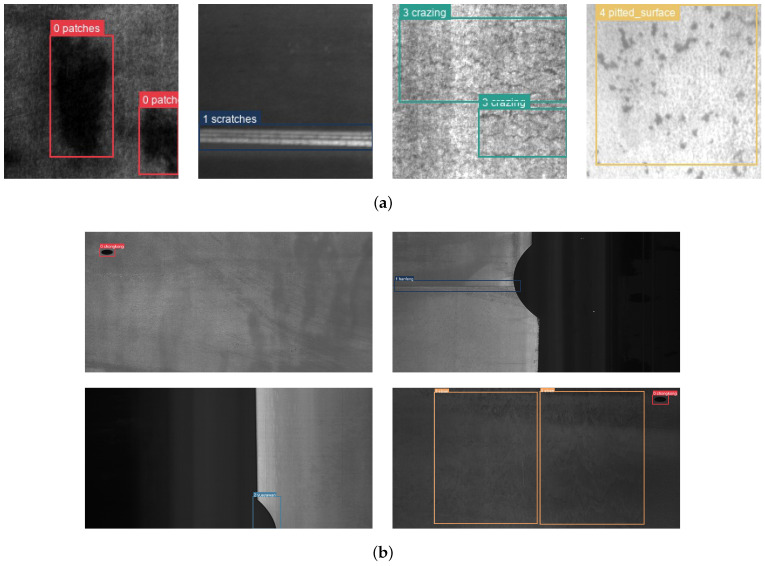
Representative defect samples from the two public datasets used in this study: (**a**) NEU-DET, (**b**) GC-10.

To evaluate MCM-YOLO for industrial metal surface defect detection and assess its performance on different public benchmarks, this article conducts experiments on two representative public datasets, namely, NEU-DET [37] and GC-10 [38]. Representative defect samples from the two datasets are shown in Figure 8.

NEU-DET is used as the primary benchmark. It contains six typical categories of hot-rolled steel strip surface defects, namely Crazing, Inclusion, Patches, Pitted Surface, Rolled-in Scale, and Scratches, with 300 images in each category. The defects in this dataset usually exhibit small scales, weak textures, blurred boundaries, and high interclass similarity, making it suitable for evaluating fine-grained defect detection performance and the effectiveness of the proposed components.

GC-10 is another public metal surface defect dataset containing ten defect categories. Compared with NEU-DET, GC-10 involves different defect categories, more diverse defect appearances, and more complex background textures. Therefore, in this study, GC-10 is adopted as an additional public benchmark for cross-dataset evaluation, which enables further examination of the proposed method under a distinct defect distribution.

### 4.2. Implementation Details

To ensure the comparability and reproducibility of the experimental results, all models were trained and evaluated under the same hardware and software environment. The experimental platform consisted of an Intel Core i9-14900K processor, 64 GB RAM, and a single NVIDIA GeForce RTX 4060 GPU. The implementation was based on Python 3.10, CUDA 12.6, and PyTorch 2.6.0.

The core training hyperparameters are listed in Table 2. The input image resolution was uniformly set to 640×640, and the batch size was set to 32. Stochastic gradient descent (SGD) was adopted as the optimizer. The initial learning rate (lr0) was set to 0.01, the learning rate decay factor (lrf) was set to 0.01, and the weight decay coefficient was set to 0.0005. Each model was trained for up to 500 epochs, with an early stopping patience of 100 epochs introduced to reduce overfitting.

A unified data augmentation strategy was adopted for all models. In addition to conventional geometric and color perturbations, Mosaic, Copy-Paste, and RandAugment were used to improve robustness against scale variations, appearance disturbances, and complex backgrounds. Mosaic was disabled during the final 10 epochs to stabilize late-stage convergence.

### 4.3. Evaluation Metrics

The models are evaluated in terms of detection accuracy and inference efficiency. For detection accuracy, Precision (*P*), Recall (*R*), average precision (AP), and mean average precision (mAP) are adopted as the core metrics, which are defined as follows:

(10) $$P=\frac{TP}{TP+FP},$$

(11) $$R=\frac{TP}{TP+FN},$$

(12) $$AP=\int_{0}^{1}P\left(R\right)\;dR,$$

(13) $$mAP=\frac{1}{N}\sum_{i=1}^{N}AP_{i},$$

where TP, FP, and FN denote the numbers of true positives, false positives, and false negatives, respectively; P(R) denotes the precision–recall function; and *N* denotes the total number of defect categories. In this article, mAP@0.5 is adopted as the basic accuracy metric, namely, the mAP computed at an intersection over union (IoU) threshold of 0.5. Meanwhile, the stricter mAP@0.5:0.95 is adopted as the comprehensive evaluation metric, which is obtained by averaging mAP over IoU thresholds ranging from 0.5 to 0.95 with a step size of 0.05 to reflect the overall detection capability of the model under higher localization accuracy requirements.

For efficiency evaluation, parameters (Params), floating-point operations (FLOPs), and frames per second (FPS) are used to measure storage overhead, theoretical computational cost, and actual inference throughput, respectively.

### 4.4. Performance Comparison

To evaluate the overall performance of MCM-YOLO in industrial metal surface defect detection, this study compares it with several representative object detection methods on the NEU-DET dataset. The compared models are selected to cover different detector paradigms and efficiency levels. Specifically, Faster R-CNN [11] is included as a representative two-stage detector, while SSD [15] and RetinaNet [16] are selected as classical one-stage detectors. EfficientDet [33] is adopted as an efficient multiscale detection framework. In addition, several lightweight YOLO-series models, including YOLOv7t [39], YOLOv8n [40], YOLOv9t [41], YOLOv10n [42], YOLOv11n [43], YOLOv12n [44], and YOLO26n [45], are included because they are widely used real-time detection baselines and are closely related to lightweight industrial deployment. RT-DETR-l [46] is further selected as a representative Transformer-based detector. The detailed comparison results in terms of detection accuracy and computational cost are listed in Table 3.

The experimental results show that MCM-YOLO achieves the highest overall accuracy-related metrics among the compared methods. Specifically, its Recall, mAP@0.5, and mAP@0.5:0.95 reach 77.6%, 80.4%, and 45.7%, respectively, all of which are the highest among the compared models. Meanwhile, MCM-YOLO maintains a low computational cost of 6.3 GFLOPs and 2.70 M parameters, indicating a favorable balance between detection accuracy and model complexity.

Compared with the baseline YOLOv11n, MCM-YOLO improves Recall from 72.4% to 77.6%, mAP@0.5 from 78.6% to 80.4%, and mAP@0.5:0.95 from 45.0% to 45.7%, while keeping the FLOPs unchanged and increasing the parameter count only slightly from 2.58 M to 2.70 M. Although the FPS decreases from 158.5 to 143.5, the model still maintains high inference throughput. This slight decrease is mainly caused by the denser multi-branch feature aggregation in Agg-BiFPN and the additional channel-spatial recalibration in MCM-SAB, which introduce extra memory access and feature-fusion operations that are not fully reflected by FLOPs. Overall, the proposed components improve the detection metrics with limited additional computational and inference cost.

The category-level detection behavior is summarized in Table 4 through per-class AP@0.5 results on the NEU-DET dataset. MCM-YOLO achieves the highest overall mAP@0.5 among the compared models and achieves competitive category-level performance on most defect classes. Specifically, MCM-YOLO obtains the highest AP on Crazing, Patches, Rolled-in Scale, and Scratches, and ties the highest AP on Inclusion. The improvements on Rolled-in Scale and Scratches suggest that the proposed framework is beneficial for defect categories with irregular textures, fine elongated structures, or background interference. Although YOLOv11n achieves a slightly higher AP on Pitted Surface, MCM-YOLO still provides the highest average category-level performance.

The comparison also shows that a larger model size does not necessarily lead to better performance in fine-grained metal surface defect detection. For example, Faster R-CNN and RT-DETR-l require much higher computational costs but obtain lower detection accuracy than MCM-YOLO. This suggests that, for datasets such as NEU-DET, where defects are small and local textures are highly sensitive, task-oriented lightweight designs for local representation, multiscale aggregation, and interface refinement may be more suitable than simply increasing model complexity.

**Figure 9:**
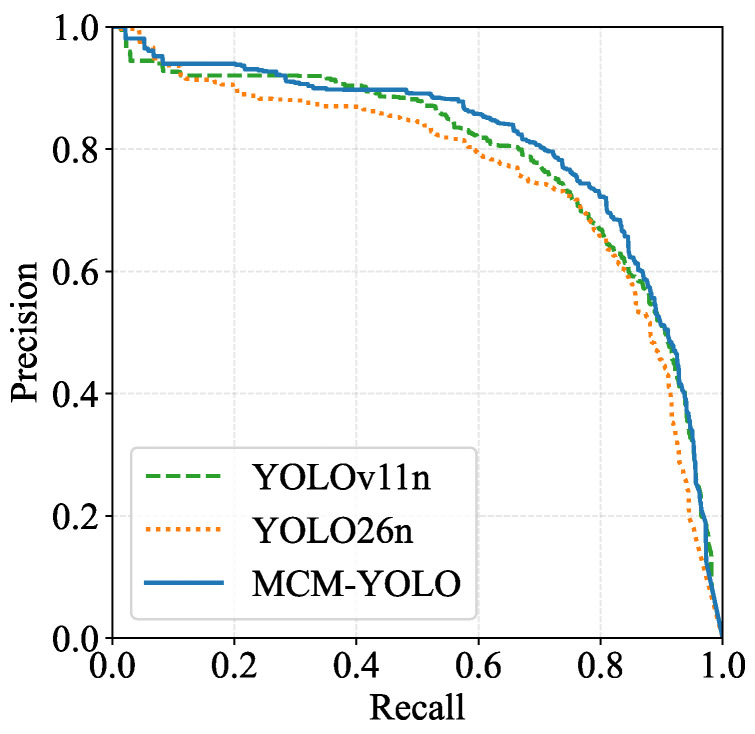
Comparison of P-R curves on the NEU-DET dataset.

Figure 9 presents the Precision–Recall (P-R) curves of YOLOv11n, YOLO26n, and MCM-YOLO on the NEU-DET dataset. The P-R curve reflects the detection balance of a model under different thresholds through the overall relationship between Precision and Recall. As shown in Figure 9, the P-R curve of MCM-YOLO generally lies outside the others and encloses the largest area. In particular, MCM-YOLO still maintains relatively high Precision in the high-Recall region. This suggests that the proposed method achieves a more favorable balance between false-positive control and defect recall under the evaluated thresholds, showing a more stable precision–recall tradeoff in complex metal surface defect detection tasks.

A more comprehensive comparison between MCM-YOLO and recent YOLO-derived variants under a similar modification setting is conducted by equipping YOLOv11n and YOLOv12n with BiFPN and representative attention modules. For fairness, ECA and CBAM are inserted at the same P4 interface position as MCM-SAB, and all variants are trained and evaluated under the same experimental settings. As shown in Table 5, although these variants introduce multiscale fusion or attention refinement with comparable computational costs, none of them outperforms MCM-YOLO. For example, YOLOv11n + BiFPN achieves 79.0% mAP@0.5, while adding CBAM or ECA does not bring further stable improvement. Similarly, the YOLOv12n-based variants equipped with BiFPN and attention modules still obtain lower mAP@0.5 than MCM-YOLO. These results suggest that simply introducing BiFPN-style fusion or generic attention modules into recent YOLO baselines is not sufficient to achieve the highest performance in this task. In contrast, the proposed task-oriented combination of C3-R2L, Agg-BiFPN, and MCM-SAB achieves higher detection metrics under a comparable computational budget.

### 4.5. Ablation Studies

#### 4.5.1. Component Ablation of MCM-YOLO

The ablation study is designed to evaluate the contribution of each proposed component and to examine the non-additive behavior observed when the enhanced backbone and strengthened neck are directly coupled. All experiments are conducted on the NEU-DET dataset and include three parts: component combination analysis, comparison of lightweight interface mediation modules, and insertion-position analysis of MCM-SAB.

Table 6 presents the performance variations obtained by progressively introducing C3-R2L, Agg-BiFPN, and MCM-SAB on top of the YOLOv11n baseline. When C3-R2L is introduced alone, mAP@0.5 increases from 78.6% to 79.2%, and mAP@0.5:0.95 increases from 45.0% to 45.2%. When Agg-BiFPN is introduced alone, mAP@0.5 further increases to 79.7%, and Recall rises from 72.4% to 74.1%. These results indicate that C3-R2L and Agg-BiFPN are individually beneficial for fine-grained local representation and cross-scale feature aggregation, respectively. However, directly combining C3-R2L and Agg-BiFPN only achieves 78.9% mAP@0.5 and 44.8% mAP@0.5:0.95, which does not produce the expected additive gains. This non-additive behavior is consistent with the FIC explanation discussed in Section 3.4, suggesting that directly coupling locally enhanced backbone features and aggregation-oriented neck inputs may introduce feature compatibility issues at the cross-module interface. After MCM-SAB is further introduced on the basis of model d, the complete model improves mAP@0.5 to 80.4%, mAP@0.5:0.95 to 45.7%, and Recall to 77.6%. This result supports the effectiveness of the proposed interface mediation design under almost unchanged computational cost.

**Figure 10:**
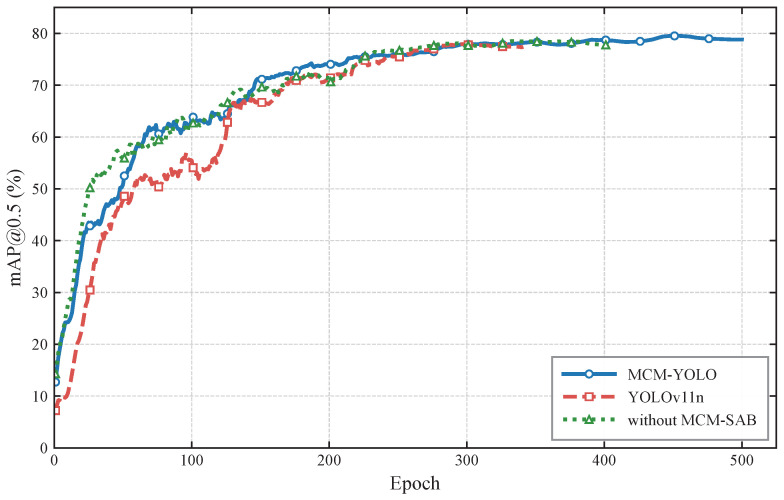
Comparison of convergence curves on the NEU-DET dataset.

The convergence behavior of YOLOv11n, model d without MCM-SAB, and complete MCM-YOLO is also compared in Figure 10. All models are trained under the same maximum epoch setting and early-stopping strategy, with the patience value set to 100. Therefore, the curves terminate at different epochs according to their actual early-stopping points rather than being manually truncated. As shown in Figure 10, the model without MCM-SAB converges normally but reaches lower validation performance than complete MCM-YOLO, suggesting that the performance gap is unlikely to be explained solely by convergence failure. After introducing MCM-SAB, MCM-YOLO achieves higher and more stable validation performance, providing additional evidence for the proposed interface mediation design.

#### 4.5.2. Comparison of Lightweight Interface Mediation Modules

The contribution of the proposed mediation design is further analyzed by comparing MCM-SAB with several lightweight interface mediation modules at the same P4 feature interface. All modules are inserted on the basis of model d, namely the model equipped with both C3-R2L and Agg-BiFPN but without MCM-SAB. As shown in Table 7, the compared modules include simple projection or alignment operations, such as 1×1 convolution and depthwise convolution (DWConv), as well as representative attention mechanisms, including Squeeze-and-Excitation (SE) [47], Convolutional Block Attention Module (CBAM) [48], ECA, and SimAM. In addition, the reversed serial order SimAM → ECA is also evaluated to examine the influence of the mediation sequence.

The results show that simply inserting a lightweight operation at the P4 interface does not necessarily lead to stable improvement. For example, the 1×1 convolution obtains only 77.7% mAP@0.5, while SE, CBAM, ECA, and SimAM also fail to achieve consistently better results than the complete MCM-SAB. DWConv improves mAP@0.5 to 79.4%, indicating that local spatial interaction at the interface may provide certain benefits. However, it still remains lower than MCM-SAB. Moreover, the reversed SimAM → ECA order achieves 78.1% mAP@0.5, which is clearly lower than the proposed ECA → SimAM sequence. This comparison suggests that first performing channel recalibration and then applying spatial/neuron-level refinement is more suitable for the current interface mediation setting. In contrast, the proposed MCM-SAB achieves the highest Recall, mAP@0.5, and mAP@0.5:0.95, reaching 77.6%, 80.4%, and 45.7%, respectively, while keeping FLOPs and parameters almost unchanged. These results provide detection-level evidence that the proposed sequential mediation strategy is more suitable than general attention modules or simple lightweight projection/alignment operations under a similar computational budget.

#### 4.5.3. Insertion Position Analysis of MCM-SAB

Table 8 compares the performance of deploying MCM-SAB at the P3, P4, and P5 feature levels individually, as well as at all levels simultaneously, to verify the rationale for the selected insertion position. The results show that deploying MCM-SAB only at the P4 level achieves the highest overall performance. From the perspective of feature flow, the P3 level mainly contains high-resolution shallow textures, whereas the P5 level carries more abstract semantic information. In contrast, the P4 level lies in the transition region between shallow details and deep semantics and serves as a critical interface where the interaction between enhanced backbone features and Agg-BiFPN aggregated features is relatively concentrated. Therefore, introducing MCM-SAB at the P4 level is more consistent with the role of interface mediation before dense multiscale aggregation. By contrast, applying refinement uniformly to all levels not only fails to bring additional benefits but may also introduce redundant constraints and interfere with normal multiscale feature flow. This result provides experimental support for using P4 as the critical refinement interface.

### 4.6. Cross-Dataset Evaluation

Experiments were also conducted on the GC-10 dataset to evaluate the performance of the proposed method on another public metal surface defect benchmark. The results are listed in Table 9. MCM-YOLO achieves the highest mAP@0.5 and mAP@0.5:0.95 among all compared models, reaching 65.1% and 33.6%, respectively. Compared with the baseline model YOLOv11n, MCM-YOLO improves mAP@0.5 from 62.7% to 65.1%, with a gain of 2.4 percentage points. Meanwhile, mAP@0.5:0.95 increases from 33.2% to 33.6%, and Recall increases from 63.3% to 64.4%. The comparison among different models also suggests that GC-10 presents a challenging detection setting due to its complex background textures and relatively large morphological variations. Under this setting, MCM-YOLO obtains the highest mAP@0.5 and mAP@0.5:0.95 values among the compared models, indicating that the proposed method maintains competitive cross-dataset benchmark performance on GC-10.

### 4.7. Grad-CAM Heatmap Analysis

**Figure 11:**
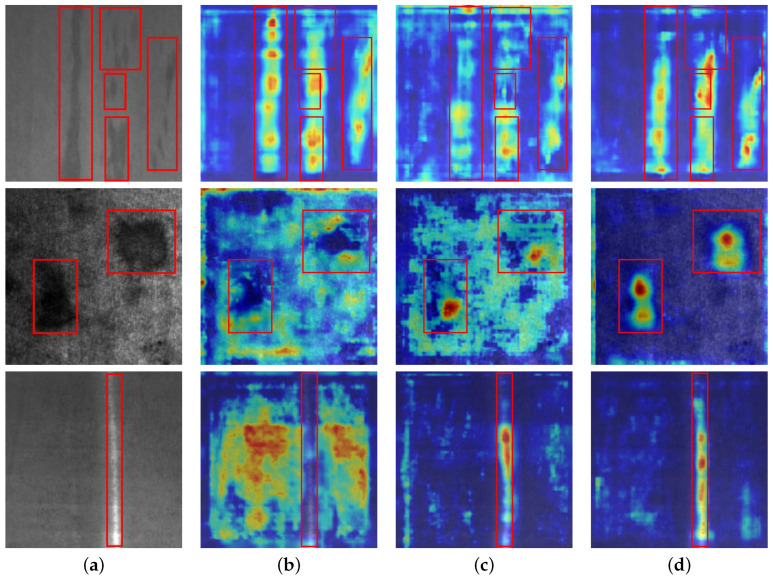
Grad-CAM visualizations of different models on representative defect samples: (**a**) input samples, (**b**) YOLOv11n, (**c**) without MCM-SAB, and (**d**) MCM-YOLO.

Feature-response differences among different models are visualized using Gradient-weighted Class Activation Mapping (Grad-CAM) heatmaps on representative defect samples. Figure 11 presents the Grad-CAM heatmaps of YOLOv11n, the model without MCM-SAB, and MCM-YOLO. Here, the model without MCM-SAB denotes the variant equipped with both the C3-R2L block and Agg-BiFPN but without the interface mediation module. The red bounding boxes indicate the ground-truth defect regions.

As shown in Figure 11, the high-response regions of YOLOv11n are relatively scattered, indicating that the baseline model has limited focus on subtle defect regions. After introducing C3-R2L and Agg-BiFPN without MCM-SAB, the heatmap responses become stronger in some areas, but response shifts and background activations can still be observed. In contrast, MCM-YOLO produces relatively stronger responses within the annotated defect regions and weaker responses in irrelevant background areas.

To reduce the subjectivity of visual inspection, two quantitative metrics were further calculated on 50 representative annotated images. Let H(i,j) denote the normalized Grad-CAM response at pixel (i,j), and let $B_{GT}$ denote the union of all ground-truth bounding-box regions in the image. The GT activation ratio is defined as

(14) $$R_{GT}=\frac{\sum_{\left(i,j\right)\in B_{GT}}H\left(i,j\right)}{\sum_{(i,j)\in \Omega }H\left(i,j\right)+\epsilon },$$

where Ω denotes the whole image region and ϵ is a small constant to avoid division by zero. This metric measures the proportion of the total Grad-CAM response falling inside the annotated defect regions. A higher $R_{GT}$ indicates that more model activation is assigned to defect-related regions.

In addition, the response contrast between the ground-truth regions and the background is calculated as

(15) $$C_{GT/BG}=\frac{\frac{1}{|B_{GT}|}\sum_{\left(i,j\right)\in B_{GT}}H\left(i,j\right)}{\frac{1}{|\Omega \setminus B_{GT}|}\sum_{\left(i,j\right)\in \Omega \setminus B_{GT}}H\left(i,j\right)+\epsilon }.$$

This metric evaluates whether the average activation inside the defect regions is stronger than that in the background. A higher $C_{GT/BG}$ indicates a clearer response contrast between defect regions and background regions.

As shown in Table 10, MCM-YOLO achieves the highest GT activation ratio and GT/BG contrast among the compared models. Specifically, the GT activation ratio increases from 0.4628±0.2308 for YOLOv11n and 0.4687±0.2401 for the model without MCM-SAB to 0.4827±0.2313 for MCM-YOLO. Similarly, the GT/BG contrast increases from 1.6541±0.9289 and 1.6589±0.9520 to 1.7141±0.9522. These results indicate that MCM-YOLO assigns relatively more activation to annotated defect regions and produces stronger contrast between defect regions and background regions. Therefore, the quantitative results provide additional support for the visual observations in Figure 11, suggesting that MCM-SAB helps enhance defect-related feature responses.

### 4.8. Qualitative Detection Visualization

**Figure 12:**
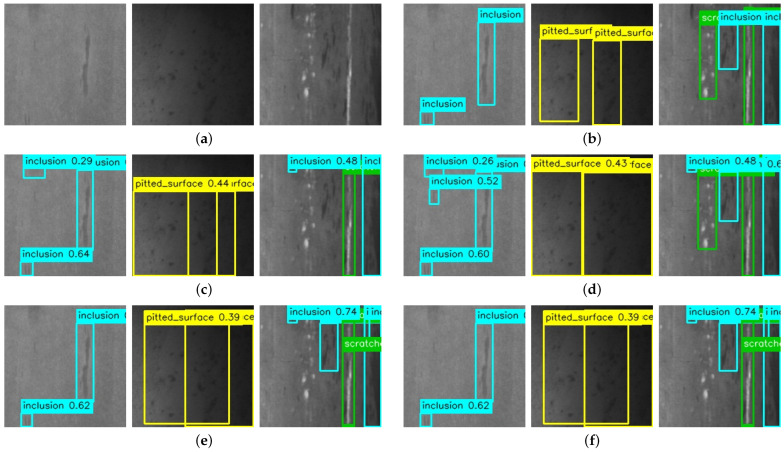
Comparison of detection results on representative defect samples: (**a**) input samples, (**b**) ground truth, (**c**) YOLOv11n, (**d**) YOLOv12n, (**e**) YOLO26n, and (**f**) MCM-YOLO.

Furthermore, Figure 12 compares the detection results of YOLOv11n, YOLOv12n, YOLO26n, and MCM-YOLO in complex metal surface defect scenarios. From the displayed samples, it can be observed that the compared lightweight models are more prone to false positives, missed detections, or less accurate localization under complex backgrounds and subtle defect conditions. For example, in the Inclusion samples, some models are affected by background textures and generate redundant detection boxes. In subtle defect samples such as Scratches, missed detections or weak responses can also be observed. In contrast, MCM-YOLO produces fewer redundant predictions, detects more subtle defects, and yields bounding boxes that better overlap with the annotated defect regions in the displayed examples. These qualitative observations are also consistent with the category-level quantitative results reported in Table 4, where MCM-YOLO achieves the highest overall mAP@0.5 and obtains better AP on most defect categories.

### 4.9. Discussion

#### 4.9.1. Failure Case Analysis

**Figure 13:**
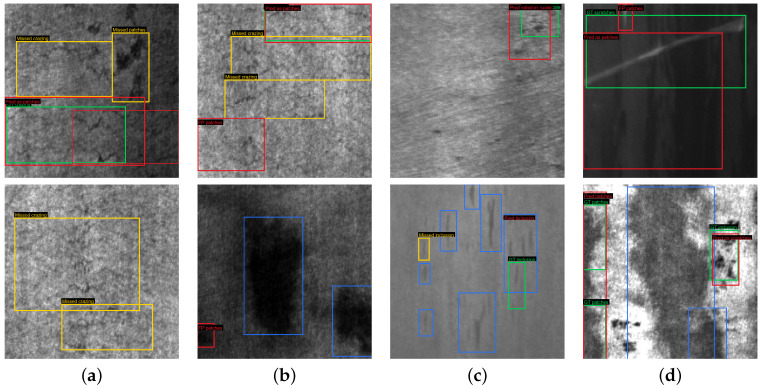
Failure cases of MCM-YOLO on the NEU-DET dataset: (**a**) missed detection, (**b**) false positive, (**c**) localization deviation, and (**d**) category confusion.

Although MCM-YOLO achieves the highest overall mAP@0.5 among the compared models and shows competitive category-level performance on most defect classes, several challenging cases can still be observed. Figure 13 presents representative failure cases of MCM-YOLO on the NEU-DET dataset. In the figure, green boxes denote ground-truth annotations, blue boxes denote correctly detected predictions, red boxes denote incorrect predictions, and yellow boxes highlight missed ground-truth defect regions.

As shown in Figure 13a, missed detections mainly occur when defect regions are weak in contrast, small in scale, or visually similar to the surrounding background. In such cases, the discriminative cues occupy only a small portion of the image and may be weakened during feature extraction and multiscale fusion. Figure 13b shows false-positive cases, where background textures, local intensity variations, or rolling marks are incorrectly detected as defects. This indicates that some non-defective texture patterns may still trigger defect-like responses when they are visually similar to real surface anomalies. Figure 13c illustrates localization deviation cases. Although the model can roughly identify the defect regions, the predicted boxes may not fully match the ground-truth boundaries. This problem is more likely to appear for defects with blurred edges, irregular shapes, or elongated structures, where the boundary between the defect and the background is not sufficiently clear. Figure 13d further shows category confusion cases, where the predicted category differs from the ground-truth label. Such errors are mainly caused by high inter-class similarity, since several metal surface defects share similar local patterns, such as dark regions, strip-like textures, or weak edge structures.

These failure cases indicate that the proposed C3-R2L, Agg-BiFPN, and MCM-SAB modules help improve defect representation and feature coordination, but they cannot fully eliminate errors caused by extremely subtle visual cues, ambiguous defect boundaries, background interference, and visually similar categories.

#### 4.9.2. Domain Shift, Rare Defects, and OOD Robustness

The experiments on GC-10 provide an additional cross-dataset evaluation on another public metal surface defect benchmark. However, both NEU-DET and GC-10 are publicly available datasets with their own specific categories, imaging conditions, and annotation protocols. Therefore, the GC-10 results should not be interpreted as strict external validation in real industrial environments. In practical industrial inspection, domain shift may arise from differences in factories, production lines, imaging devices, illumination conditions, material surfaces, manufacturing processes, and operating environments. These factors can introduce scene-specific interference that is not fully covered by public benchmark datasets.

From this perspective, robustness to rare defects, unseen textures, and out-of-distribution (OOD) samples remains an important open issue. Rare defects are usually under-represented during training, and unseen defect patterns may not share the same texture distribution as the available training samples. In addition, changes in surface material, illumination, camera parameters, or production conditions may cause background textures to differ significantly from those in the training data. Under such circumstances, a detector may rely partly on scene-specific visual cues rather than transferable defect-related information, which can reduce its reliability in previously unseen industrial environments.

Recent studies in intelligent fault diagnosis provide useful but indirect perspectives on this issue. For example, category-invariant disentangled feature learning has been investigated for domain generalization in machine fault diagnosis, suggesting that separating category-invariant information from domain-sensitive variations can help improve representation stability under changing industrial conditions [49]. Although this idea was originally developed for machinery fault diagnosis rather than visual surface defect detection, it is conceptually relevant to the present task because defect-related cues should ideally be distinguished from variations caused by background textures, imaging settings, and production environments. This perspective also indicates that improving detection accuracy on public benchmarks is not equivalent to fully learning domain-invariant defect representations.

In addition, OOD-oriented data augmentation has been explored for rotating machinery fault detection under zero-faulty data settings [50]. Such studies highlight the potential value of constructing auxiliary abnormal or OOD samples when real fault data are scarce or unavailable. This is also relevant to surface defect detection, where rare defect categories and unseen texture patterns are often difficult to collect in sufficient quantities. Nevertheless, the present study does not explicitly implement domain-invariant representation learning, explicit feature disentanglement, or OOD-oriented data augmentation. Therefore, the findings of this work should be interpreted within the scope of lightweight metal surface defect detection on two public benchmark datasets. The current results support the effectiveness of MCM-YOLO in improving the accuracy–efficiency tradeoff and cross-dataset benchmark performance, but they do not by themselves establish robustness to unseen production lines, rare defect categories, or strict OOD samples. Future work will further investigate domain-invariant defect representation, defect-related feature disentanglement, OOD-oriented data augmentation, and validation on independently collected production-line datasets.

## 5. Conclusions

This article proposed a lightweight defect detection framework, termed MCM-YOLO, for industrial metal surface defect detection. Built upon YOLOv11, the proposed method enhances fine-grained local representation in the backbone through the C3-R2L block and strengthens multiscale feature aggregation in the neck via Agg-BiFPN. In addition, considering that directly coupling the enhanced backbone and strengthened neck may lead to performance degradation, this work describes this phenomenon as FIC and introduces MCM-SAB to alleviate potential feature incompatibility at the critical interface. Experimental results on the NEU-DET and GC-10 datasets show the effectiveness of the proposed framework. On NEU-DET, MCM-YOLO achieves 80.4% mAP@0.5 and 45.7% mAP@0.5:0.95 with 6.3 GFLOPs and 2.70 M parameters. On GC-10, it obtains 65.1% mAP@0.5 and 33.6% mAP@0.5:0.95. These results indicate that the proposed method helps improve the representation, aggregation, and coordination of metal defect features while maintaining a competitive accuracy–efficiency tradeoff.

Although the proposed method achieved promising results on two public metal surface defect datasets, several issues still deserve further investigation. First, since both NEU-DET and GC-10 are publicly available benchmarks, the current results should be interpreted as evaluations on public datasets rather than strict validation of external industrial generalization. The effectiveness of MCM-YOLO under real production-line domain shifts, such as different imaging devices, illumination conditions, material surfaces, and manufacturing environments, still needs to be further examined. Second, although the FIC explanation is supported by ablation studies and feature-level observations, its underlying mechanism requires more systematic analysis in broader detection scenarios. In addition, rare defects, unseen textures, and out-of-distribution samples remain challenging for lightweight defect detectors. Future work will further investigate domain-invariant representation learning, defect-related feature disentanglement, and OOD-oriented data augmentation to improve the robustness and practical generalization of the proposed framework.

## Figures and Tables

**Table 1:** Functional roles of the proposed components in MCM-YOLO.

Component	Location	Design Purpose	Functional Contribution
C3-R2L	Backbone	Enhances fine-grained local representation for weak-texture, blurred-boundary, and small-scale defects.	Introduces lightweight hierarchical local multiscale modeling to improve sensitivity to subtle texture variations and local anomalous patterns.
Agg-BiFPN	Neck	Strengthens cross-scale feature interaction and multi-source feature aggregation.	Builds denser bidirectional feature fusion paths with adaptive weighted aggregation to improve the coordination between shallow details and deep semantics.
MCM-SAB	P4 interface between the enhanced backbone and strengthened neck	Performs lightweight interface mediation before dense multiscale aggregation.	Sequentially applies ECA-based channel recalibration and SimAM-based spatial/neuron-level refinement to improve the compatibility of interface features.

**Table 2:** Training hyperparameters.

Hyperparameter	Configuration
Epochs	500
Batch size	32
Input size	640
Optimizer	SGD
lr0	0.01
lrf	0.01
Weight decay	0.0005

**Table 3:** Comparison of detection performance and efficiency of different models on the NEU-DET dataset.

Model	P (%)	R (%)	mAP@0.5 (%)	mAP@0.5:0.95 (%)	FLOPs (G)	Params (M)	FPS
Faster R-CNN	71.0	71.5	75.6	36.8	369.8	136.79	14.2
SSD	74.9	58.9	71.8	35.9	61.3	24.28	79.4
RetinaNet	66.1	62.2	65.8	32.5	146.9	36.43	30.2
EfficientDet	68.5	65.5	68.4	34.5	11.6	6.56	47.2
YOLOv7t	69.8	58.5	69.5	35.3	13.9	6.23	87.9
YOLOv8n	77.9	71.8	78.4	44.6	8.1	3.01	174.8
YOLOv9t	74.1	74.0	78.7	45.2	7.6	1.97	108.4
YOLOv10n	71.6	72.3	76.3	43.7	8.2	2.70	151.8
YOLOv11n	75.1	72.4	78.6	45.0	6.3	2.58	158.5
YOLOv12n	72.9	72.4	75.6	43.9	6.3	2.56	125.6
YOLO26n	76.2	70.2	75.8	43.5	5.2	2.38	152.6
RT-DETR-l	65.9	68.5	69.2	38.8	103.5	31.99	61.7
MCM-YOLO	76.2	77.6	80.4	45.7	6.3	2.70	143.5

**Table 4:** Per-class AP@0.5(%) comparison on the NEU-DET dataset.

Model	Crazing	Inclusion	Patches	Pitted Surface	Rolled-In Scale	Scratches	mAP@0.5 (%)
YOLOv11n	48.7	84.3	92.1	87.7	65.9	93.0	78.6
YOLOv12n	42.3	86.1	92.7	79.8	59.1	93.4	75.6
YOLO26n	39.9	80.3	89.8	85.2	66.2	93.3	75.8
RT-DETR-l	26.4	77.2	87.3	80.8	55.6	88.0	69.2
MCM-YOLO	49.8	86.1	93.0	87.4	70.6	95.3	80.4

**Table 5:** Comparison with enhanced YOLOv11n and YOLOv12n variants.

Model	P (%)	R (%)	mAP@0.5 (%)	mAP@0.5:0.95 (%)	FLOPs (G)	Params (M)	FPS
YOLOv11n + BiFPN	76.7	74.4	79.0	45.0	6.2	2.54	150.6
YOLOv11n + BiFPN + CBAM	75.5	74.3	79.0	44.5	6.2	2.57	148.1
YOLOv11n + BiFPN + ECA	74.1	77.5	78.7	45.4	6.2	2.54	148.7
YOLOv12n + BiFPN	72.9	74.2	77.8	44.6	6.3	2.54	122.7
YOLOv12n + BiFPN + CBAM	71.7	71.6	76.6	44.0	6.3	2.57	117.9
YOLOv12n + BiFPN + ECA	69.7	75.5	77.4	44.9	6.3	2.54	118.9
MCM-YOLO	76.2	77.6	80.4	45.7	6.3	2.70	143.5

**Table 6:** Ablation results of MCM-YOLO.

Model	C3-R2L	Agg-BiFPN	MCM-SAB	P (%)	R (%)	mAP@0.5 (%)	mAP@0.5:0.95 (%)	FLOPs (G)	Params (M)
a				75.1	72.4	78.6	45.0	6.32	2.58
b	✓			77.2	73.4	79.2	45.2	6.26 (−0.06)	2.54 (−0.04)
c		✓		78.6	74.1	79.7	44.7	6.34 (+0.02)	2.75 (+0.17)
d	✓	✓		75.2	73.7	78.9	44.8	6.28 (−0.04)	2.70 (+0.12)
e	✓	✓	✓	76.2	77.6	80.4	45.7	6.28 (−0.04)	2.70 (+0.12)

**Table 7:** Comparison of lightweight interface mediation modules.

Interface Module	P (%)	R (%)	mAP@0.5 (%)	mAP@0.5:0.95 (%)	FLOPs (G)	Params (M)
1×1 Conv	78.0	71.6	77.7	44.3	6.3	2.72
DWConv	75.0	74.1	79.4	45.1	6.3	2.70
SE	77.1	71.2	78.0	44.7	6.3	2.70
CBAM	76.4	73.4	78.6	44.2	6.3	2.74
ECA	75.1	76.0	78.7	45.1	6.3	2.70
SimAM	75.2	73.5	78.1	44.6	6.3	2.70
SimAM → ECA	76.2	73.1	78.1	45.2	6.3	2.70
ECA → SimAM (MCM-SAB)	76.2	77.6	80.4	45.7	6.3	2.70

**Table 8:** Comparison of different insertion positions of MCM-SAB.

Insertion Level	P (%)	R (%)	mAP@0.5 (%)	mAP@0.5:0.95 (%)	FLOPs (G)	Params (M)
None	75.2	73.7	78.9	44.8	6.3	2.70
P3	78.9	71.6	78.9	45.3	6.3	2.70
P4	76.2	77.6	80.4	45.7	6.3	2.70
P5	77.2	71.0	78.0	43.8	6.3	2.70
All (P3/P4/P5)	74.2	71.8	76.7	41.5	6.3	2.70

**Table 9:** Cross-dataset evaluation results on the GC-10 dataset.

Model	P (%)	R (%)	mAP@0.5 (%)	mAP@0.5:0.95 (%)
Faster R-CNN	63.0	59.6	59.7	24.6
SSD	63.3	50.6	56.7	23.3
RetinaNet	67.6	45.3	48.7	20.6
EfficientDet	70.3	53.5	55.6	24.4
YOLOv7t	72.0	61.5	64.6	29.0
YOLOv8n	70.9	60.4	64.6	33.3
YOLOv9t	71.8	61.0	64.7	32.8
YOLOv10n	71.2	57.7	62.0	32.2
YOLOv11n	62.9	63.3	62.7	33.2
YOLOv12n	65.1	60.9	63.4	32.0
YOLO26n	68.8	60.4	62.9	33.3
RT-DETR-l	56.3	61.4	58.4	29.5
MCM-YOLO	68.9	64.4	65.1	33.6

**Table 10:** Quantitative analysis of Grad-CAM responses in ground-truth regions.

Model	GT Activation Ratio ↑	GT/BG Contrast ↑
YOLOv11n	0.4628±0.2308	1.6541±0.9289
Without MCM-SAB	0.4687±0.2401	1.6589±0.9520
MCM-YOLO	0.4827±0.2313	1.7141±0.9522

## Data Availability

The public datasets used in this study are available from their original sources. The NEU-DET dataset is available at http://faculty.neu.edu.cn/songkechen/zh_CN/zdylm/263270/list/ (accessed on 1 June 2026), and the GC-10 dataset is available at https://github.com/lvxiaoming2019/GC10-DET-Metallic-Surface-Defect-Datasets (accessed on 1 June 2026).

## References

[1] Ren Z., Fang F., Yan N., Wu Y. (2022). State of the art in defect detection based on machine vision. Int. J. Precis. Eng. Manuf.-Green Technol..

[2] Shi W., Cao J., Zhang Q., Li Y., Xu L. (2016). Edge computing: Vision and challenges. IEEE Internet Things J..

[3] Ma Y., Lu C., Sinopoli B., Zeng S. (2020). Exploring edge computing for multitier industrial control. IEEE Trans. Comput.-Aided Des. Integr. Circuits Syst..

[4] Ma Y., Yin J., Huang F., Li Q. (2024). Surface defect inspection of industrial products with object detection deep networks: A systematic review. Artif. Intell. Rev..

[5] Gao Y., Lin J., Xie J., Ning Z. (2021). A real-time defect detection method for digital signal processing of industrial inspection applications. IEEE Trans. Ind. Inform..

[6] Li L., Ota K., Dong M. (2018). Deep learning for smart industry: Efficient manufacture inspection system with fog computing. IEEE Trans. Ind. Inform..

[7] Lee S., Chang L.-M., Skibniewski M.J. (2006). Automated recognition of surface defects using digital color image processing. Autom. Constr..

[8] Choi D.-C., Jeon Y.-J., Kim S.H., Moon S., Yun J.P., Kim S.W. (2017). Detection of pinholes in steel slabs using Gabor filter combination and morphological features. ISIJ Int..

[9] Tsai D.-M., Chen M.-C., Li W.-C., Chiu W.-Y. (2012). A fast regularity measure for surface defect detection. Mach. Vis. Appl..

[10] Aminzadeh M., Kurfess T. (2015). Automatic thresholding for defect detection by background histogram mode extents. J. Manuf. Syst..

[11] Ren S., He K., Girshick R., Sun J. (2017). Faster R-CNN: Towards real-time object detection with region proposal networks. IEEE Trans. Pattern Anal. Mach. Intell..

[12] He K., Gkioxari G., Dollár P., Girshick R. Mask R-CNN. Proceedings of the IEEE International Conference on Computer Vision (ICCV).

[13] Wei R., Song Y., Zhang Y. (2020). Enhanced faster region convolutional neural networks for steel surface defect detection. ISIJ Int..

[14] Wang H., Li M., Wan Z. (2022). Rail surface defect detection based on improved Mask R-CNN. Comput. Electr. Eng..

[15] Liu W., Anguelov D., Erhan D., Szegedy C., Reed S., Fu C.-Y., Berg A.C. (2016). SSD: Single Shot MultiBox Detector. Computer Vision–ECCV 2016.

[16] Lin T.-Y., Goyal P., Girshick R., He K., Dollár P. Focal loss for dense object detection. Proceedings of the IEEE International Conference on Computer Vision (ICCV).

[17] Redmon J., Divvala S., Girshick R., Farhadi A. You only look once: Unified, real-time object detection. Proceedings of the IEEE Conference on Computer Vision and Pattern Recognition (CVPR).

[18] Ma G., Wu M., Wu Z., Yang W. (2021). Single-shot MultiBox detector- and building information modeling-based quality inspection model for construction projects. J. Build. Eng..

[19] Yang Z., Liu Y. (2025). A steel surface defect detection method based on improved RetinaNet. Sci. Rep..

[20] Zhou Y., Zhao Z. (2025). MPA-YOLO: Steel surface defect detection based on improved YOLOv8 framework. Pattern Recognit..

[21] Shen M., Liu Y., Chen J., Ye K., Gao H., Che J., Wang Q., He H., Liu J., Wang Y. (2024). Defect detection of printed circuit board assembly based on YOLOv5. Sci. Rep..

[22] Wang Y., Yun W., Xie G., Zhao Z. (2025). YOLO-WAD for small-defect detection boost in photovoltaic modules. Sensors.

[23] Fan Z., Zhao Y., Liu C., Qiu J. (2025). DB-YOLO: A dual-branch parallel industrial defect detection network. Sensors.

[24] Chen L., Guo C., Wu X., Xu H., Chen S., Lin J. (2026). Confidence–gradient reweighting and lightweight feature enhancement algorithm for steel surface defect detection. Sci. Rep..

[25] Gao L., Zhang J., Yang C., Zhou Y. (2022). Cas-VSwin transformer: A variant Swin transformer for surface-defect detection. Comput. Ind..

[26] Liu H., Chen C., Hu R., Bin J., Dong H., Liu Z. (2024). CGTD-Net: Channel-wise global Transformer-based dual-branch network for industrial strip steel surface defect detection. IEEE Sens. J..

[27] Chen X., Zhang X., Shi Y., Pang J. (2025). HCT-Det: A high-accuracy end-to-end model for steel defect detection based on hierarchical CNN–Transformer features. Sensors.

[28] Shang H., Sun C., Liu J., Chen X., Yan R. (2023). Defect-aware transformer network for intelligent visual surface defect detection. Adv. Eng. Inform..

[29] Papa L., Russo P., Amerini I., Zhou L. (2024). A survey on efficient vision transformers: Algorithms, techniques, and performance benchmarking. IEEE Trans. Pattern Anal. Mach. Intell..

[30] Khanam R., Hussain M. (2024). YOLOv11: An overview of the key architectural enhancements. arXiv.

[31] Lin T.-Y., Dollár P., Girshick R., He K., Hariharan B., Belongie S. Feature pyramid networks for object detection. Proceedings of the IEEE Conference on Computer Vision and Pattern Recognition (CVPR).

[32] Liu S., Qi L., Qin H., Shi J., Jia J. Path aggregation network for instance segmentation. Proceedings of the IEEE/CVF Conference on Computer Vision and Pattern Recognition (CVPR).

[33] Tan M., Pang R., Le Q.V. EfficientDet: Scalable and efficient object detection. Proceedings of the IEEE/CVF Conference on Computer Vision and Pattern Recognition (CVPR).

[34] Wang Q., Wu B., Zhu P., Li P., Zuo W., Hu Q. ECA-Net: Efficient channel attention for deep convolutional neural networks. Proceedings of the IEEE/CVF Conference on Computer Vision and Pattern Recognition (CVPR).

[35] Yang L., Zhang R.-Y., Li L., Xie X. (2021). SimAM: A simple, parameter-free attention module for convolutional neural networks. Proceedings of the 38th International Conference on Machine Learning (ICML), Virtual, 18–24 July 2021.

[36] Gao S.-H., Cheng M.-M., Zhao K., Zhang X.-Y., Yang M.-H., Torr P.H.S. (2021). Res2Net: A new multi-scale backbone architecture. IEEE Trans. Pattern Anal. Mach. Intell..

[37] He Y., Song K., Meng Q., Yan Y. (2020). An end-to-end steel surface defect detection approach via fusing multiple hierarchical features. IEEE Trans. Instrum. Meas..

[38] Lv X., Duan F., Jiang J.-J., Fu X., Gan L. (2020). Deep metallic surface defect detection: The new benchmark and detection network. Sensors.

[39] Wang C.-Y., Bochkovskiy A., Liao H.-Y.M. YOLOv7: Trainable bag-of-freebies sets new state-of-the-art for real-time object detectors. Proceedings of the IEEE/CVF Conference on Computer Vision and Pattern Recognition (CVPR).

[40] Jocher G., Chaurasia A., Qiu J. (2023). Ultralytics YOLOv8. https://docs.ultralytics.com/models/yolov8/.

[41] Wang C.-Y., Yeh I.-H., Liao H.-Y.M. (2024). YOLOv9: Learning what you want to learn using programmable gradient information. Computer Vision–ECCV 2024.

[42] Wang A., Chen H., Liu L., Chen K., Lin Z., Han J., Ding G. (2024). YOLOv10: Real-time end-to-end object detection. arXiv.

[43] Jocher G., Qiu J. (2024). Ultralytics YOLO11. https://docs.ultralytics.com/models/yolo11/.

[44] Tian Y., Ye Q., Doermann D. (2025). YOLOv12: Attention-centric real-time object detectors. arXiv.

[45] Jocher G., Qiu J. (2026). Ultralytics YOLO26. https://docs.ultralytics.com/models/yolo26/.

[46] Zhao Y., Lv W., Xu S., Wei J., Wang G., Dang Q., Liu Y., Chen J. DETRs beat YOLOs on real-time object detection. Proceedings of the IEEE/CVF Conference on Computer Vision and Pattern Recognition (CVPR).

[47] Hu J., Shen L., Sun G. Squeeze-and-excitation networks. Proceedings of the IEEE/CVF Conference on Computer Vision and Pattern Recognition (CVPR).

[48] Woo S., Park J., Lee J.-Y., Kweon I.S. (2018). CBAM: Convolutional block attention module. Computer Vision–ECCV 2018.

[49] Chen Z., Wu J., Deng Z., Huang H.-Z. (2026). Learning category-invariant disentangled features for domain generalization in machine fault diagnosis. IEEE/ASME Trans. Mechatron..

[50] Chen Z., Huang H.-Z., Deng Z., Wu J. (2025). Shrinkage Mamba relation network with out-of-distribution data augmentation for rotating machinery fault detection and localization under zero-faulty data. Mech. Syst. Signal Process..

